# EpCAM-claudin-tetraspanin-modulated ovarian cancer progression and drug resistance

**DOI:** 10.1080/19336918.2020.1732761

**Published:** 2020-02-24

**Authors:** Zehra Tavsan, Hülya Ayar Kayalı

**Affiliations:** a Chemistry Department, The Graduate School of Natural and Applied Science, Dokuz Eylül University, İzmir, Turkey; b Izmir Biomedicine and Genome Center, Izmir, Turkey; c Chemistry Department, Science Faculty, Dokuz Eylül University, Izmir, Turkey; d International Biomedicine and Genome Institute, Dokuz Eylül University, Izmir, Turkey

**Keywords:** Ovarian cancer, cell adhesion, EpCAM, claudins, tetraspanins, palmitoylation

## Abstract

Alterations of cell adhesion are involved in cancer progression, but the mechanisms underlying the progression and cell adhesion have remained poorly understood. Focusing on the complex between EpCAM, claudins and tetraspanins, we described a sequence of events by which of the molecules associate each other in ovarian cancer. The interactions between molecules were evaluated by immunoprecipitations and then immunoblotting. To identify the effects of complex formation on the ovarian cancer progression, the different types of ovarian cancer cell lines were compared. In this study, we report the identification of the EpCAM-claudin-4 or −7-CD82 complex in the ovarian cancer progression and metastasis in vitro. Additionally, we demonstrated palmitoylation and intra- or extra-cellular regions are critically required for the complex formation. These results represent the first direct evidence for the link between the dynamism of cell adhesion molecules and ovarian cancer progression.

## Introduction

Epithelial ovarian cancer (EOC) is the fifth commonest cancer and the leading cause of gynaecological cancer death among women. The non-specific nature of symptoms caused to diagnostic delay and the most women patient presented with the metastasized (FIGO stages III–IV) disease at the time of diagnosis [1]. About 85% to 90% of malignant ovarian tumours were derived from the ovarian surface epithelium (OSE) or inclusion cysts [2]. Studies have shown that the EOC has a complex differentiation mechanism and is composed of diverse tumour groups with distinctive morphologic and molecular genetic properties [3].

The adhesion of epithelial cells to their neighbours and the extracellular matrix is mediated by the different types of junctional complexes and cell adhesion molecules. These complexes are also engaged in the signal transmissions. Recent studies focused on to gain insights into the cancer hallmarks about the role of cell adhesion during the transformation of epithelial cells. Tumour cells need cooperative activities between individual cell adhesion molecules for the invasion to surrounding tissue and metastasis to distant organs. One of the intercellular cell adhesion molecules is a type I, transmembrane, 39–42 kDa glycoprotein, Epithelial cell adhesion molecule (EpCAM) which aberrant expression is characteristic during and after malignant transformation [4,5]. Also, Claudins are among the most important tight junction proteins which help to regulate paracellular permeability and mediate cell adhesion. In general, claudin-1 and −7 were downregulated in oesophageal cancer [6], but upregulated in others [7,8]. The most common claudin-3 and −4 were usually upregulated in cancers. The ability of individual cells to differentiate their plasma membrane to form specialized domains such as tetraspanin-enriched microdomains (TEMs) with distinct proteins is crucial for many cellular biological processes [9,10]. Many studies have found correlations between tetraspanins and cancer progression [11,12]. There is ample evidence for the complex of cell adhesion molecules, EpCAM and claudin-7, and a tetraspanin, promoting colorectal cancer progression [13]. The complex between these molecules, rather than the individual molecules, was responsible for the cancer progression and also drug resistance.

The gains about diagnosis and treatment of ovarian cancer are rather modest, and the rigorous and focused assessment of individual gene products contribution to tumorigenesis in the molecular pathogenesis of ovarian cancer remains a need to better understand so new molecular therapeutic targets for early detection and treatment have been identified. The functional consequences and the underlying molecular mechanisms of the complex between EpCAM, claudins and tetraspanins have not yet been explored in the ovarian cancer progression. Starting from the hypothesis that the complex between EpCAM, claudins and tetraspanins might influence ovarian cancer progression and chemoresistance, we evaluated whether and which of the molecules associate each other. Furthermore, the indicated interactions were analysed in the tumour samples which were obtained from xenograft ovarian cancer animals.

## Materials and methods

### Cell lines and cell culture

The cell lines used were normal ovarian surface epithelial (OSE), A2780, OVCAR–3, SKOV–3 and A2780cis. Normal ovarian surface epithelial (OSE) cell line was purchased from Abm-Good. A2780, OVCAR–3, SKOV–3 and cisplatin-resistant A2780cis ovarian cancer cell lines were obtained from the European Collection of Authenticated Cell Cultures (ECACC). The cells were resuscitated from liquid nitrogen stocks and cultured for less than 2 months before reinitiating culture from the same passage. ECACC and Abm-Good had authenticated the cell lines.

### Cell growth conditions

A2780, OVCAR–3 and A2780cis, SKOV–3 and OSE cell lines were grown in RPMI, McCoy’s 5A and Prigrow I growth mediums, respectively. All growth mediums contain 10% foetal bovine serum (FBS) and 1% penicillin-streptomycin. The cells were incubated in a CO_2_ incubator, conditioned to 37 °C and 5% CO_2_ levels.

### The preparation of cell and tissue lysates

The cells were lysed in the lysis buffer (50 mM Tris, pH 8.0, 150 mM NaCl) including the appropriate detergent (1% Triton X-100, Brij 96 and CHAPS). After sonication and incubation on ice, lysates were centrifuged at 13,300 rpm for 20 minutes at 4 °C. Total proteins were purified from snap frozen tumours after homogenization in 50 mM Tris-HCl (pH 8.0), 150 mM NaCl, 1% Triton X-100. Lysates were cleared by centrifuging at 13,300 rpm for 20 min. The supernatants were used for western blotting and immunoprecipitation. The protein levels were determined with BCA assay as indicated in the manufacturer’s instructions.

### Immunoprecipitation

The immune complexes consisting of protein-antibody-Protein A/G beads in the supernatants were collected by centrifugation at 13,300 rpm for 20 minutes at 4 °C. The cleared pellets were incubated in SDS-PAGE loading buffer at 95 °C for 20 minutes and maintained in the solubilized form by centrifugation.

### Western blotting

The supernatants were mixed with SDS-PAGE loading buffer and loaded onto gels. The proteins were fractioned by SDS-PAGE and electroblotted onto the membranes. After blocking, membranes were incubated with the primary antibodies of EpCAM (Abcam-ab71916;1:1000), claudin-1 (Thermo-374900;1:250), claudin-3 (Thermo-PA5-37526;1:250), claudin-4 (Abcam-ab53156;1:750), claudin-7 (Thermo-PA5-23689;1:250), E-cadherin (Cell Signalling-3195 S;1:1000), PKCα (Santacruz-sc-208;1:250), PKCβ1 (Santacruz-sc-209;1:250), GAPDH (Santacruz-sc-25778;1:2500), CD82 (Tspan-27) (Thermo-PA5-20356;1:200) and CD9 (Tetraspanin-29) (Cell Signalling-13174;1:1000). The membranes were screened by HRP-conjugated secondary antibodies.

### Immunofluorescence analysis

Cells were grown on glass coverslips and ﬁxed in 4% paraformaldehyde (PFA). After permeabilization and blocking, the cells were incubated with the antibodies against studied molecules and then with secondary antibodies conjugated with Alexa Fluor 488 and 594. The nucleus was stained with DAPI. Cells were mounted upside down on the microscope slide and the localization of studied molecules was assessed with immunofluorescence microscopy.

### Inhibition of palmitoylation, cross-linking and cholesterol depletion

70–80% confluent cells were incubated 50 μM of palmitoylation inhibitor, 2-bromopalmitate (2-BP) for 20 hours at 37 °C. Membrane permeable and impermeable cross-linking agents, DSP and DTSSP were treated at 1 mM for 30 minutes at 37 °C.

### Xenograft animal studies

Six- to eight-week-old female BALB/c nude mice were provided by the Izmir Biomedicine and Genome Centre (Izmir, Turkey). The animals were housed in microisolator cages in a pathogen-free animal bio-safety level-2 facility at 22 ± 2°C. Human SKOV-3 cells (5x10^6^ cells/mice) were injected intraperitoneally (i.p.) into the immunodeficient mice (n = 6). Animals were assigned based on their genotype, age and sex matched. No randomization was used. All procedures involving the use and care of mice were approved ethically and scientifically by the university in compliance with the Practice Guidelines for Laboratory Animals of Turkey.

### Statistical analysis

Data were analysed for n = 3 experiments. No samples or animals have been excluded from the analysis.

## Results

The first goal of this study was to examine the changes in the levels of cell adhesion molecules involved in ovarian cancer progression and metastasis, and to determine whether and which of the molecules associate each other. The difference between the characters of ovarian cancer cells lines demonstrated the functions of complex in ovarian cancer progression and metastasis.

### The expressions of cell adhesion molecules alter in the ovarian cancer progression

**Figure 1.**
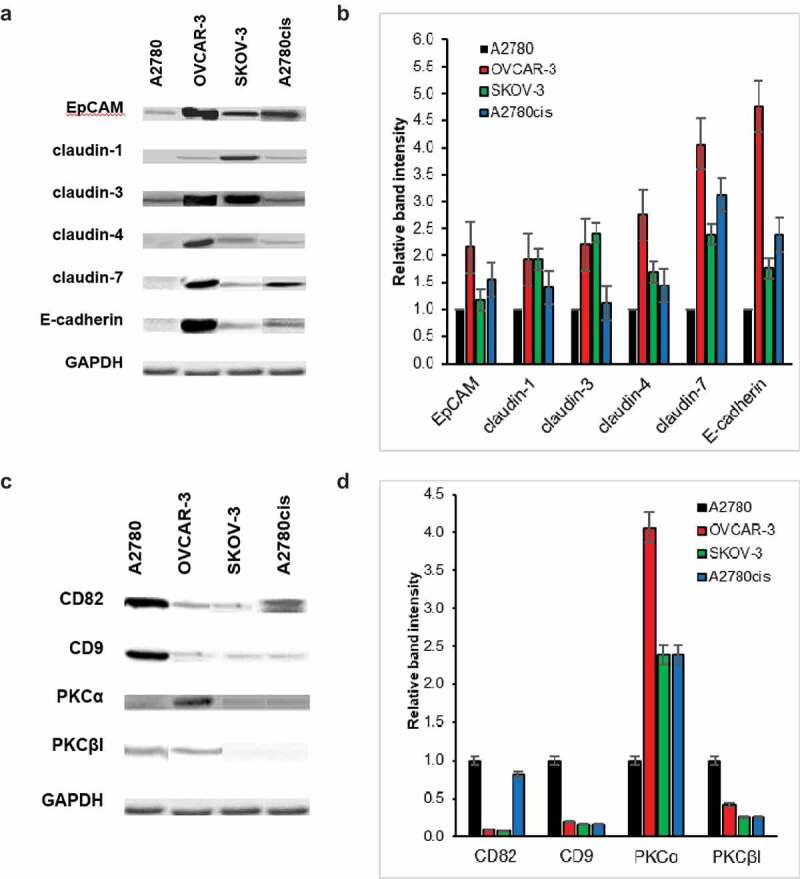
Protein levels of EpCAM, claudin isoforms and E-cadherin, tetraspanins alter in ovarian cancer progression and chemoresistance. A and C A2780, OVCAR-3, SKOV-3 and A2780cis cells were lysed in 1% Triton X-100 containing lysis buffer. After SDS-PAGE and transfer to nitrocellulose membranes, blots were incubated with the indicated antibodies. GAPDH was used as a control. B and D Bar graphs show the relative band intensity of EpCAM, claudin-1, −3, −4 and −7, E-cadherin, CD82 and CD9 in A2780, OVCAR-3, SKOV-3 and A2780cis cells (n = 3; mean ± SEM)

The expression patterns of EpCAM, claudins, E-cadherin and tetraspanins were investigated compared within ovarian cancer cell lines, A2780, OVCAR-3, SKOV-3 and A2780cis (Figure 1). As shown in Figure 1(a), the levels of EpCAM and claudin isoforms were also low in A2780 cells which were differentiated from normal ovarian surface epithelial cells. When examined at the protein level, it was found that EpCAM and claudin isoforms were upregulated in OVCAR–3, SKOV–3 and A2780cis cells compared with A2780 cells (Figure 1(a,b). Western blot results of ovarian surface epithelial (OSE) cells showed that they did not express claudin isoforms and E-cadherin (data not shown). Similar to EpCAM and claudins expressions, E-cadherin was expressed in OVCAR-3, SKOV-3 and A2780cis cells while it was very low in A2780. CD82 and CD9 were downregulated in OVCAR-3 and SKOV-3 cells compared to A2780 (Figure 1(c,d)).

### EpCAM-claudin-tetraspanin complex forms through ovarian cancer progression

Coexpression of EpCAM, claudins and tetraspanin, rather than the expressions of the individual molecules at low or high levels, may be correlated with the ovarian cancer progression. We speculated that the molecules might interact, and their concerted activity might promote tumour progression. To support this hypothesis, we first evaluated whether and which of these molecules associate each other using coimmunoprecipitation.

**Figure 2.**
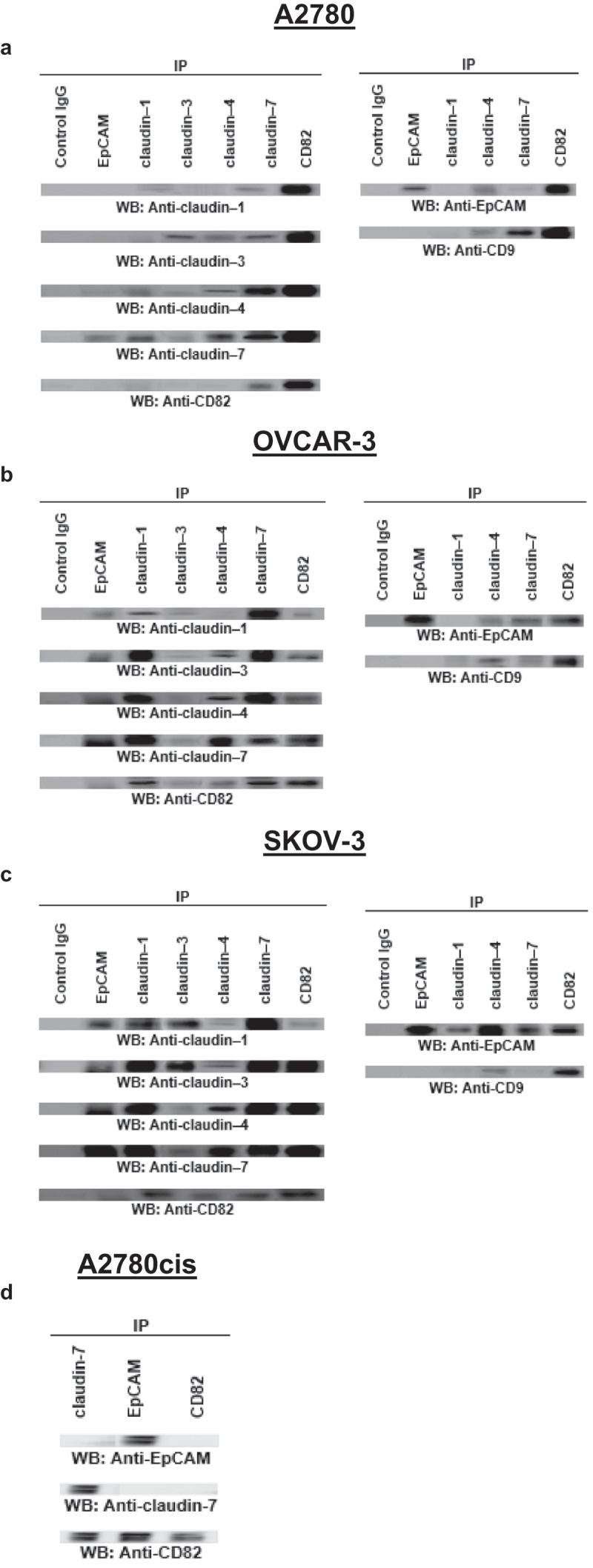
EpCAM, claudins and tetraspanins interact each other in ovarian cancer cells. The cells were lysed in 1% Triton X-100 containing buffer. Lysates were precipitated with the indicated antibodies of studied molecules. A2780 (a), OVCAR-3 (b), SKOV-3 (c) and A2780cis (d) cells were used in this experiment (n = 3)

Figure 2 showed whether and which of EpCAM, claudin-1, −3, −4, and −7, CD82 and CD9 interacted each other in A2780, OVCAR-3 and SKOV-3 cells. EpCAM and CD9 coimmunoprecipitated with claudin-4 and vice-versa. Claudin-3, −4 and −7 showed homo- and heterophilic interactions with claudin-4. Claudin-7 immunoprecipitates contained EpCAM, claudin-1, −3, −4 and −7, CD82 suggesting claudins interacted at the low levels with the other studied molecules, although claudin levels are so low as to be absent. EpCAM, claudin-1, −3, −4 and −7 and CD9 precipitated in the CD82 immunoprecipitates. Also, it was shown that CD82, located in the tetraspanin-enriched microdomains (TEMs) acted as a scaffold to regulate the interactions. EpCAM coimmunoprecipitated with claudin-1, −3, −4, −7 and CD82 in OVCAR-3 cells and vice-versa (Figure 2(b)). Heterophilic interactions between claudin-4 and claudin-7 were determined. The amounts of claudin-1 were at the low levels in claudin-4 immunoprecipitates while claudin-3 was significantly higher. Similar to A2780, CD82 collected all the studied molecules in CD82 immunoprecipitates of OVCAR-3 cells. As seen in Figure 2(c), in SKOV-3 cells, EpCAM precipitated in claudin-1, −3, −4, −7 and CD82 immunoprecipitates. Claudin-1 interacted with both claudin-4 and −7, and vice-versa. Also, claudin-4 immunoprecipitates contained claudin-7. In addition to EpCAM and claudins, homophilic and heterophilic interactions were shown between claudin isoforms. Claudin-1 did not coimmunoprecipitate or hardly immunoprecipitate with claudin-4 and −7 in A2780 cells. Instead, immunoprecipitates of claudin-1 in OVCAR-3 and SKOV-3 cells contained claudin-4 and −7, together with upregulated expressions of claudin-4 and −7. The coimmunoprecipitations of claudin-1, −4 and −7 with CD82 demonstrated that these complexes were also located in TEMs.

### EpCAM-claudin-tetraspanin complex influences drug resistance

Coexpression was observed in the primary ovarian A2780 cancer cells and also the metastasizing OVCAR-3 and SKOV-3 cancer cells. Thus, we also asked, whether chemoresistance of human ovarian cancer was so correlated with EpCAM, claudin-4 and −7, and CD82 coexpression/complex formation. The immunoprecipitation experiments in the cisplatin-resistant A2780 cell line, A2780cis the pointed towards a significant contribution of EpCAM/claudin-7/CD82 complex to apoptosis resistance (Figure 2(d)).

### EpCAM, claudins and tetraspanin colocalize in both metastasizing and drug-resistant ovarian cancer cells

**Figure 3.**
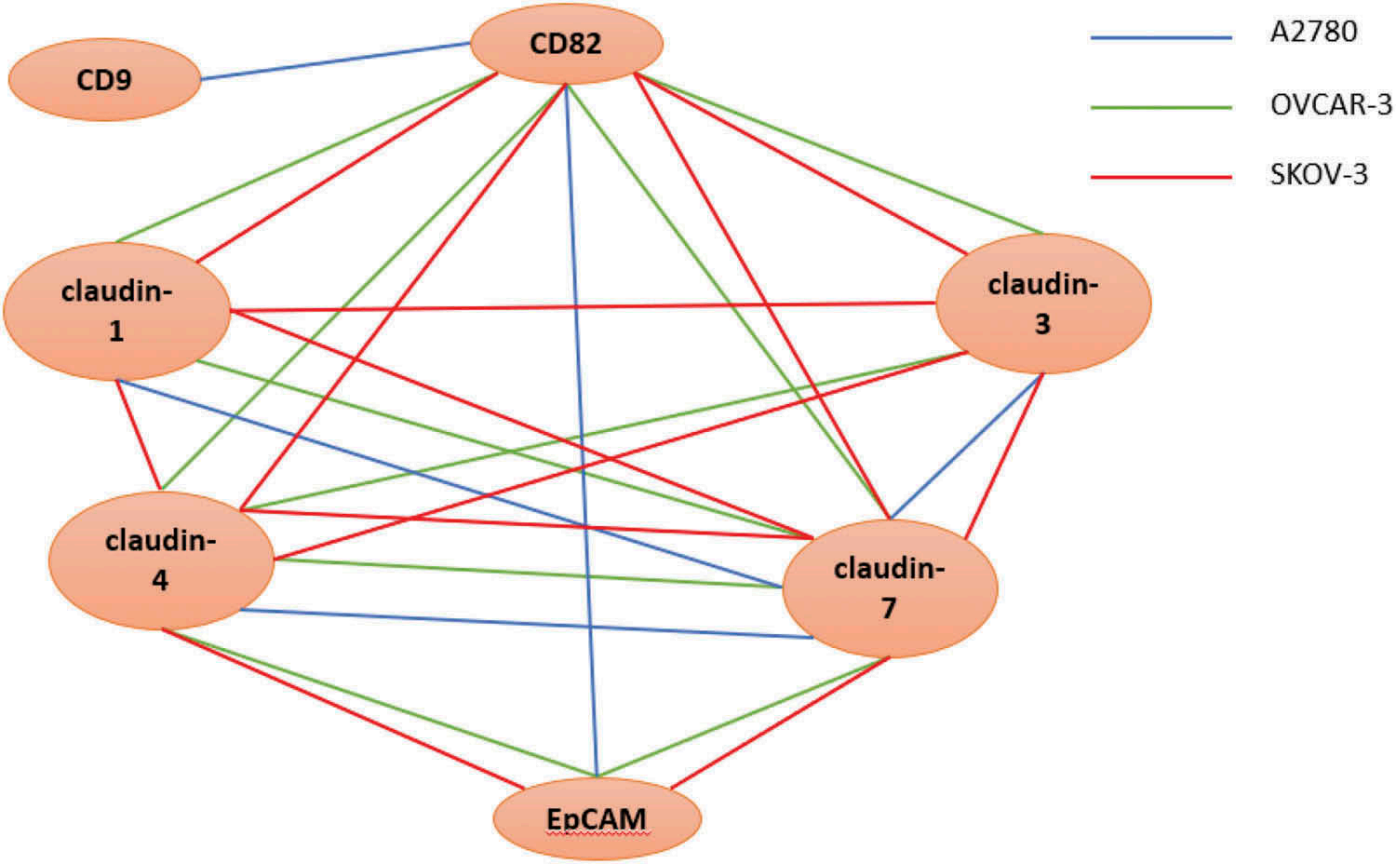
The illustration of possible interactions in A2780, OVCAR-3 and SKOV-3 cells. The blue, green and red lines show the possible complexes between the molecules in A2780, OVCAR-3 and SKOV-3 cells, respectively

**Figure 4.**
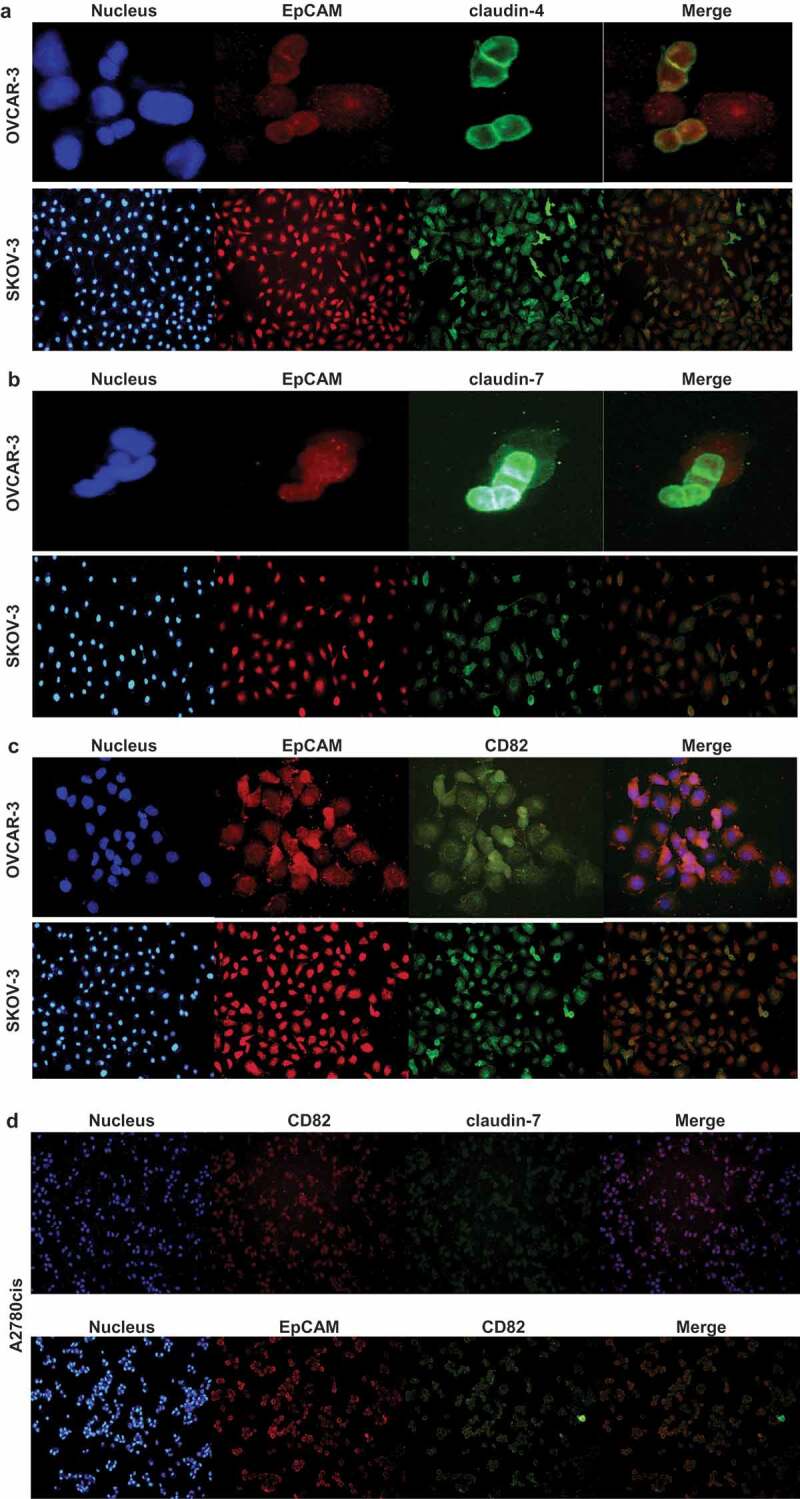
EpCAM/claudin-4, EpCAM-claudin-7 and EpCAM/CD82 colocalize in OVCAR-3 and SKOV-3 cells. OVCAR-3 and SKOV-3 cells were double-stained with anti-EpCAM/anti-rabbit IgG-Alexa 594 and anti-claudin-4/anti-mouse IgG-Alexa 488 (a), anti-claudin-7/anti-mouse IgG-Alexa 488 (b), anti-CD82/anti-mouse IgG-Alexa 488 (c). A2780cis cells were double-stained with anti-CD82/anti-rabbit IgG-Alexa 594 and anti-claudin-7/anti-mouse IgG-Alexa 488 or anti-EpCAM/anti-rabbit IgG-Alexa 594 and anti-CD82/anti-mouse IgG-Alexa 488 (d). Staining was analysed using a fluorescence microscope (magnifications, 10, 20 and 40X; 80 µm scale bar) and digital overlays. Yellow staining indicates colocalization

In addition to immunoprecipitation, fluorescence colocalization claims the physical association of molecules which interact with each other. As seen in Figure 4(a,b) and c, EpCAM associated with claudin-4 and −7 in OVCAR-3 and SKOV-3 cells irrespective of whether the lines expressed CD82, showing low-level expression of CD82 sufficed for colocalization. Colocalization of EpCAM and CD82 verified these findings. In A2780cis cells, EpCAM and claudin-7 colocalized with CD82 (Figure 4(d)). The results obtained by coimmunoprecipitation were confirmed by colocalization and showed that claudin-4 and −7, and CD82 interacted with EpCAM in OVCAR-3 and SKOV-3 cells (Figure 3).

### The palmitoylation and the interaction within intra- or extracellular domains of claudins and tetraspanin affect the complexes

**Figure 5.**
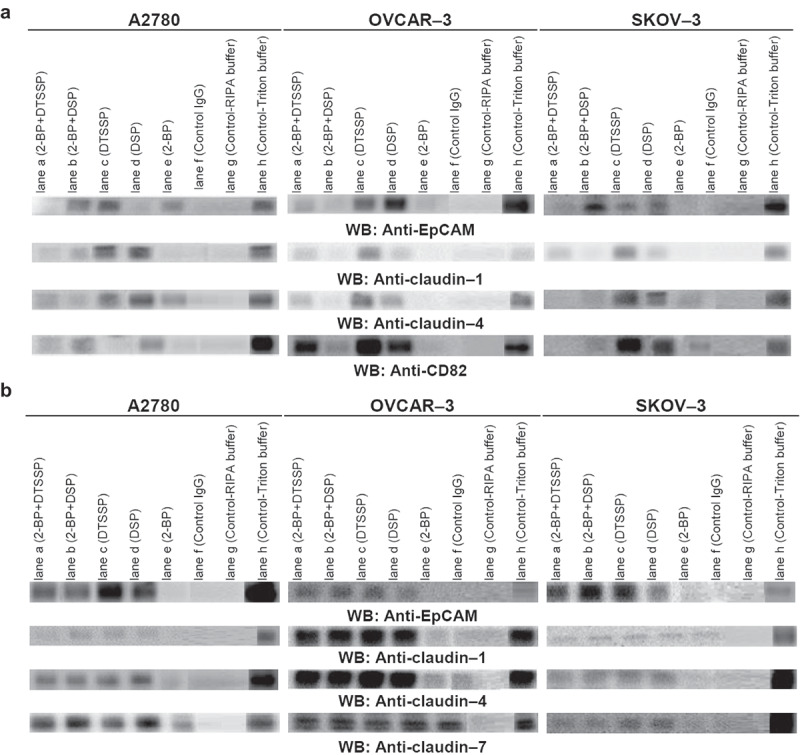
Palmitoylation statues of cell adhesion molecules and the interactions by intra- or extracellular residues modulate the assembly of complexes. A2780, OVCAR-3 and SKOV-3 cells were treated with palmitoylation inhibitor, 2-BP, the membrane-permeable cross-linker DSP or the membrane-impermeable cross-linker DTSSP, and conjugated. Lysates were precipitated with anti-CD82 (a) and claudin-7 (b)

In metastasizing and cisplatin-resistant ovarian cancer cells, claudin-4 or-7/EpCAM/CD82 complex has been detected and the question arose whether the interactions were direct or indirect. After the disruption of secondary and indirect interactions with the lysis in Triton X-100 containing a buffer, claudin-4 and −7 were recovered in EpCAM and CD82 precipitates, defining EpCAM, claudin-4 or −7 and CD82 as the direct binding partners. Furthermore, to verify direct interaction between EpCAM, claudins and tetraspanins, we used chemical crosslinking. A2780, OVCAR-3 and SKOV-3 cells were treated with the cross-linkers DSP and DTSSP (Figure 5). In both conditions which DSP and DTSSP were used for crosslinking of intracellular and extracellular cysteine domains, respectively (Figure 5, lanes c and d), the interactions of EpCAM, claudin-1 and −4 with CD82 were conserved in OVCAR-3 and SKOV-3 cells, even lysed in the RIPA buffer compared with untreated control samples. The results showed convincing evidence of their close proximity in OVCAR-3 and SKOV-3 cells by both intracellular and extracellular interaction domains. In contrast to OVCAR-3 and SKOV-3 cells, the interactions between EpCAM and CD82 in A2780 cells were lost when lysed in the RIPA buffer, even DSP treatment (Figure 5, lane d) while EpCAM maintained association with CD82 under relatively harsh detergent conditions of RIPA buffer only when cells were treated with DTSSP (Figure 5, lane c). These results suggested that CD82 and EpCAM interact via extracellular domains; vice versa, claudin-1 and −4 interactions with CD82 need intracellular domains. Similarly, with the results of OVCAR-3 and SKOV-3, the interactions of claudin-1 and −4 with CD82 were conserved in A2780 cells treated with DSP (lane d), but they were lost under DTSSP treated conditions (Figure 5, lane c).

Many transmembrane proteins, not only tetraspanins but also undergo palmitoylation [14–17]. But, how the palmitoylation affects the assembly of complexes or specialized sites in the plasma membrane is still controversy. As seen in Figure 5, after treatment of intact A2780, OVCAR-3 and SKOV-3 cells with 2-BP which is widely used to inhibit protein palmitoylation, the interactions of EpCAM, claudin-1 and −4 with CD82 were examined in CD82 immunoprecipitates. The interactions of EpCAM with CD82 in A2780, OVCAR-3 and SKOV-3 cells were greatly destroyed compared with untreated control samples, lysed in RIPA buffer run in parallel (Figure 5, lane e). In addition, CD82 also did not coimmunoprecipitate with claudin-1 and −4 in OVCAR3 and SKOV-3 cells, treated with 2-BP (Figure 5, lane e). Similarly, after 2-BP treatment, EpCAM, claudin-1 and −4 molecules were not determined in the claudin-7 immunoprecipitates of A2780, OVCAR-3 and SKOV-3 cells (Figure 5, lane e). The results showed that palmitoylation regions may be directly responsible for the interactions between EpCAM, claudin isoforms and CD82.

Both crosslinking of intra- and extracellular domains and palmitoylation may be responsible in the stable interactions of studied molecules because palmitoylation by membrane-bound DHHC proteins affects the close proximity of interaction between molecules for promotion of stable membrane associations. Therefore, to evaluate the effects of palmitoylation-mediated membrane association on the crosslinking domains, A2780, OVCAR-3 and SKOV-3 cells were treated with first 2-BP and then DSP or DTSSP (Figure 5). When palmitoylation was firstly inhibited, the effects of DSP and DTSSP on the CD82-EpCAM interactions reversed in A2780 cells (Figure 5, lanes a and b). The results demonstrated that palmitoylation domains were more important than the intracellular and extracellular interaction domains. 2-BP plus DSP treatment (Figure 5, lane b) in A2780 cells reduced the band density of CD82-claudin-1 and CD82-claudin-4 interactions compared with single 2-BP (lane e) and DSP treatments (Figure 5, lane d). In contrast, the interactions between EpCAM and claudin-1 with CD82 of A2780 cells increased after 2-BP plus DTSSP treatment (Figure 5, lane a). While CD82-claudin-1 interactions were lost in OVCAR-3 cells treated with 2-BP, 2-BP plus DSP and 2-BP plus DTSSP treatments have recovered the interactions. Similarly, claudin-4 levels in CD82 immunoprecipitates were not obviously altered in OVCAR-3 cells after 2-BP plus DSP (lane b) and 2-BP plus DTSSP treatments (Figure 5, lane a). DSP and DTSSP treatment after inhibition of palmitoylation did not recover the interactions of EpCAM, claudin-1 and −4 with CD82, because of the significant disruption of the interaction with 2-BP treatment. It showed that palmitoylation state may be force interaction domains into close interaction proximity. In A2780, OVCAR-3 and SKOV-3 cells treated with 2-BP plus DSP and DTSSP, the levels of EpCAM, claudin-1 and −4 associated with claudin-7 did not alter compared with the cells treated with DSP and DTSSP, suggesting that the interaction domains instead of palmitoylation domains may be important for the formation and stabilization of the interactions.

### In vivo EpCAM-claudin-tetraspanin complex formation

**Figure 6.**
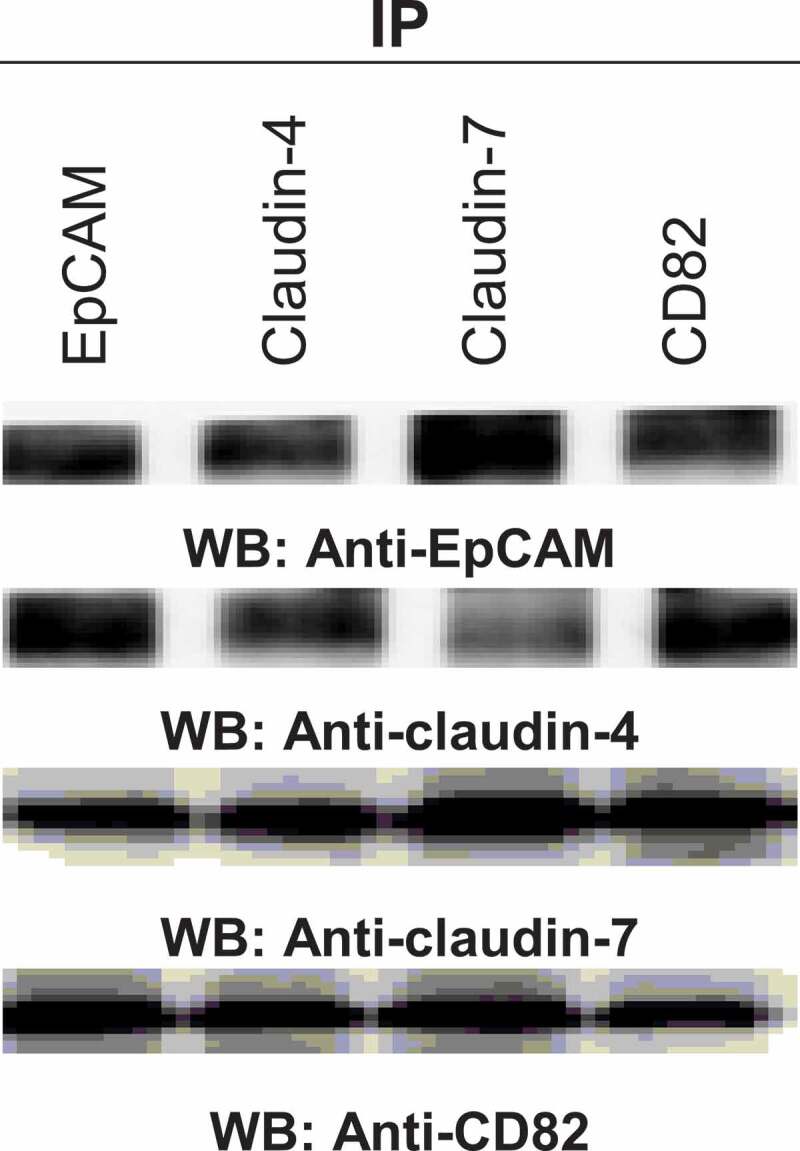
The interactions in tumours of xenograft ovarian cancer mice. After 4 weeks, the tumours were taken from the animals and total proteins were purified from snap frozen tumours by homogenization in 50 mM Tris-HCl pH 8.0, 150 mM NaCl, 1% Triton X-100. The indicated molecules were immunoprecipitated and analysed by western blotting

EpCAM, claudin-4 and −7, and CD82 coexpression correlated with the metastasis and drug resistance in ovarian cancer. To reassure the *in vivo* relevance of complex formation, EpCAM, claudin-4 and −7, and CD82 coimmunoprecipitations were evaluated in the harvested tumour tissues of xenograft ovarian cancer mice. The tumours in 5 × 10^6^ cell-injected nude mice were metastasized on the liver surface, diaphragm and colon through the peritoneal cavity. In line with the *in vitro* findings, we noted *in vivo* experiments that EpCAM, claudin-4 and −7, and CD82 formed a complex in TEM (Figure 6). Analysing the coexpression of these molecule pairs significantly strengthened the difference between metastatic tumours and primary tumours (data not shown).

## Discussion

The adhesion of epithelial cells to their neighbours and the extracellular matrix is mediated by different types of junctional complexes. In addition to the mediation of adhesion, these complexes are also engaged in the signal transmissions. It has become increasingly apparent that the dynamism of adhesion, established intra- and intercellular interactions with cell adhesion molecules have a vital role in maintaining cell integrity and tissue homoeostasis which unintended loss cause to the differentiation of cells, invasion and metastasis of cancer cells.

Similar to our findings which prove the role of EpCAM through ovarian cancer metastasis, Van der Gun et al. (2011) also showed A2780 cell line as EpCAM-negative line, SKOV-3 with an intermediate EpCAM and OVCAR-3 with a high EpCAM expression [18]. In a similar manner with our results, claudin-3 and claudin-4 upregulated in 90% of the ovarian cancer cells [19,20]. A few studies observed the claudin-7 overexpression in the ovarian cancer [21–23]. All results demonstrated that claudin overexpression was responsible for ovarian cancer progression. However, in OVCAR–3 cells, the upregulated expressions of EpCAM and all studied claudins which are epithelial markers, compared to A2780 and SKOV-3 cells defined that the mesenchymal ovarian cancer cells gained their epithelial characters back to adhere onto the peritoneum after metastasis from tumour capsules and also showed both epithelial and mesenchymal features. Uncontrolled upregulated expression of the molecules such as EpCAM and claudins in the metastasizing OVCAR-3 and SKOV-3 cells may contribute to increased risk because cell adhesions mechanically link the cells and are critical for the controlling differentiation and invasion. As a member of another junctional complex in the epithelial cells, E-cadherin expression in the ovarian carcinoma effusion fluids significantly was higher than the patient-matched primary carcinomas [24]. Furthermore, higher expression levels of E-cadherin in OVCAR-3 than SKOV-3 cells demonstrated that OVCAR-3 cells underwent MET in contrast to SKOV-3 cells. The absence of cell adhesion molecules’ expression in ovarian surface epithelial (OSE) cell line may facilitate a primitive differentiation state which occurs in OSE cells covering over the ovary and this feature helps cells becoming highly migratory to ﬁll the large wounds that are generated during oocyte release [1]. Agarwal et al. also showed that claudin-3 and −4 isoforms were not expressed by OSE cells [25]. Besides cell adhesion molecules and junctional complexes between cell adhesion and cytoskeleton proteins, tetraspanins form specialized domains with several distinct proteins and lipids, and tetraspanin-enriched microdomains (TEM) are crucial for many cell biological processes, including cell adhesion, signalling, migration and cell division [9,10]. Houle et al. (2002) and Schindl et al. (2001) showed CD82 and CD9 downregulation in the ovarian cancer [26,27]. The results of our experiments and other studies proved the cancer metastasis suppressor role of CD82 and CD9, clinically connected to the progression, invasion, and metastasis of various malignancies [28,29]. Similarly, CD82 expression was frequently downregulated or lost in poorly differentiated cancers or at the advanced stage of cancers [30,31]. CD9 is important for microvesicle biogenesis and sorting of the cargo proteins related with cancer progression [32]. In the last years, tetraspanin8 was identified therapeutic target in the ovarian cancer [33].

We assumed that the complex between EpCAM, claudins and tetraspanins, rather than the individual molecules, might promote tumour progression. The following observations supported this hypothesis: a) claudin-4 and −7 coimmunoprecipitated, albeit weakly with EpCAM in A2780 cells. Instead, in the metastatic OVCAR-3 and SKOV-3 cells which expressed EpCAM, claudin-4 and −7 higher than A2780, claudin-4 and −7 strongly coimmunoprecipitated with EpCAM and vice-versa. b) CD82 immunoprecipitates contained claudin-4 and claudin-7, higher in OVCAR-3 and SKOV-3 cells. The results showed that CD82, even at low levels supported the formation of EpCAM-claudins complexes in TEMs. On the other hand, this complex has been seen in ionic detergent lysates and insoluble phase of non-ionic detergent lysates, and it demonstrated TEM-localized EpCAM/claudins/tetraspanins complex (data not shown). In addition, these complexes were also stable in the soluble phase of the strong non-ionic detergent, Triton X-100 lysates. The findings confirmed that claudin-4 and −7 directly and strongly associated with EpCAM and suggested that claudin-4 and −7–associated EpCAM only, or at least predominantly, becomes recruited towards CD82. CD82, even at low levels in OVCAR-3 and SKOV-3 tethered the other interacted molecules. The colocalization of EpCAM and claudins with CD82 showed the involvement in the complex formation. Alternatively, colocalization of the molecules, even expressed at the low levels, could be a result of enrichment in the membrane microdomains that facilitate proximity. Ladwein et al. noted in the pancreatic adenocarcinoma tumours that CD44v6, CO-029, EpCAM, and claudin-7 formed the complexes in TEMs [34]. Also, the complexes were detected in colorectal cancer, and expression of the complex inversely correlated with disease-free survival [13]. Okada et al. suggested the involvement of EpCAM, together with CD44v6 and claudin-7 as well as ALDH1 in the aggressive anaplastic thyroid carcinoma [35]. Besides ovarian cancer progression, the complex of EpCAM/claudin-7/CD82 involved in the apoptosis resistance [13]. Consistent with our results, ovarian cancers with high levels of EpCAM had significantly much lower responsive rates after first-line chemotherapy [36]. Also, claudin-7 knockdown cells displayed decreased tumour growth and impaired migration and motility, due to the recruitment of EpCAM with claudin-7 [37]. Taken together, ovarian cancer frequently expresses a complex of EpCAM, claudin-4 or −7 and CD82 that is located in TEMs. The complex, rather than the individual molecules, promoted ovarian progression and involved in the apoptosis resistance. In addition to the studied tetraspanins and cell adhesion molecules, the complex of other tetraspanin CD151 and cell adhesion molecule α3β1 integrin suppressed ovarian cancer progression repressing the other signalling pathways [38].

To identify the directly associated proteins, we used these strategies that involve (i) covalent cross-linking of exposed cysteines, (ii) partial inhibition of protein palmitoylation to expose membrane-proximal cysteines, iii) partial disruption of cholesterol. Using these experiments, we discovered the direct protein-protein interactions between EpCAM, claudin-4 or −7 and CD82. At the first step, CD82 and claudin-7 immunoprecipitates of membrane-permeable cross-linker DSP-treated lysates contained EpCAM, which provided pronouncedly evidence for the direct complex between EpCAM, claudin-4 or −7 and CD82. Besides, the complex has been seen after OVCAR-3 and SKOV-3 cells had been treated with the membrane-impermeable cross-linker DTSSP. Thus, the cytoplasmic tails and extracellular domains of the molecules contribute to EpCAM/claudin-4 or −7/CD82 complex. Surprisingly, in A2780 cells CD82 interacted with EpCAM via extracellular domains while needed intracellular domains for the claudin interactions. Similarly, EpCAM and claudin-7 associated via intracellular cytoplasmic tails in pancreatic adenocarcinoma and colorectal cancer cells [34,39]. LC-MS/MS analysis in CD9 immunoprecipitates of crosslinker DTME-treated lung cancer cells identified direct interactions with claudin-1 although other claudins (claudin-2, −3, −4, −5, and −7) associated to a much lesser extent [17]. At the second step, we showed palmitoylation could provide stability by contributing to a more ordered state whereas protein-protein interactions probably determined the specificity of tetraspanin associations [40]. The treatment with 2-BP of intact A2780, OVCAR-3 and SKOV-3 cells greatly destroyed the interactions of EpCAM, claudin and CD82. Claudin-14 underwent palmitoylation [41], whereas CD9 can associate with claudins and stabilize them when they are not localized in the tight junctions [17]. In contrast, some studies indicated that removal of palmitoylation sites from tetraspanins does not disrupt primary protein-protein associations, but the loss of palmitoylation reduced the secondary tetraspanin associations which are caused to the impaired cell signalling and altered cell morphology [42,43].

Cell culture experiments with ovarian cancer cells which have different characters showed that coexpression of EpCAM, claudins and tetraspanin was related to ovarian cancer progression and also cisplatin resistance. Thus, we asked whether the coexpressions may provide a diagnostic and/or prognostic factor. In line with our hypothesis that to mimic metastatic stage in ovarian cancer patients, we generated xenograft ovarian cancer mouse model. The immunoprecipitation experiments of xenograft tumours demonstrated similar results with cell line experiments. Similarly, the metastasizing gastrointestinal tumours frequently expressed a complex composed of the tetraspanin D6.1A and CD44v6, EpCAM, claudin-7 [34,44]. The evaluation of colorectal cancer and liver metastasis patients showed that complex formation of the EpCAM, claudin-7, CO-029, and CD44v6 was correlated with clinical data [13]. The involvement of EpCAM, CD44v6 and claudin-7 in the thyroid cancer progression has been showed [35].

Collectively, we showed a novel mechanism, responsible for ovarian cancer progression and chemotherapeutic drug resistance *in vitro* and *in vivo*. Our findings assigned EpCAM-claudin-4/-7-CD82 complex, and posttranslational modifications and also cysteine residues had a significant role for assembly of complexes.

## Data Availability

The datasets used and/or analysed during the current study are available from the corresponding author upon reasonable request.
